# Thoracic endovascular aortic repair with a right thoracotomy approach

**DOI:** 10.1186/s13019-022-01778-x

**Published:** 2022-03-04

**Authors:** Hideki Tanioka, Takanori Shibukawa, Keiji Iwata

**Affiliations:** Department of Cardiovascular Surgery, Sakai City Medical Center, 1-1-1 Ebaraji-cho, Nishi-ku, Sakai, 593-8324 Japan

**Keywords:** TEVAR (thoracic endovascular aortic repair), Right thoracotomy, Meandering of the aorta

## Abstract

**Background:**

The common femoral artery is usually the preferred access route for thoracic endovascular aortic repair (TEVAR). However, if access from the common femoral artery is challenging, other routes must be considered. We report a case of TEVAR performed by approaching the descending thoracic aorta with a right thoracotomy and using the descending thoracic aorta as an access route.

**Case presentation:**

A 70-year-old female was diagnosed with a descending thoracic aortic aneurysm (65 mm in diameter), a thoracoabdominal aneurysm (54 mm in diameter), and an abdominal aortic aneurysm (49 mm in diameter). Since the patient had severe chronic obstructive pulmonary disease, one-stage replacement of the thoracoabdominal aortic aneurysm was contraindicated and TEVAR on the descending aorta was selected. A strong tortuous section of the aorta—from the descending aorta to the abdominal aorta—hampered endovascular access to the site from the common femoral artery. A TEVAR approach from the abdominal aorta was also considered; however, an abdominal aortic aneurysm and a transverse colon loop stoma from an earlier surgery presented challenges to this technique. We chose to access the descending thoracic aorta with a thoracotomy from the right 6th intercostal space for TEVAR, because the access route that is not affected by the meandering of the aorta is considered to be the descending aorta with a right thoracotomy. The patient’s postoperative course was uneventful after the stent graft was placed. No complications were detected with postoperative contrast-enhanced computed tomography (CT).

**Conclusions:**

Our findings suggest that TEVAR can be performed by approaching the descending aorta from a right thoracotomy, if variations of vascular anatomy interfere with the more commonly used femoral artery approach.

## Background

The common femoral artery is the preferred access route for thoracic endovascular aortic repair (TEVAR). However, use of this route can sometimes be challenging, such as in cases of smaller artery diameter from the common iliac artery to the common femoral artery, poorly characterized arterial wall, or a strong tortuous aorta. In such cases, another access route for TEVAR must be used [1]. We report a case of a TEVAR performed by approaching the descending aorta with a right thoracotomy and using the descending thoracic aorta as an access route.

## Case presentation

**Fig. 1:**
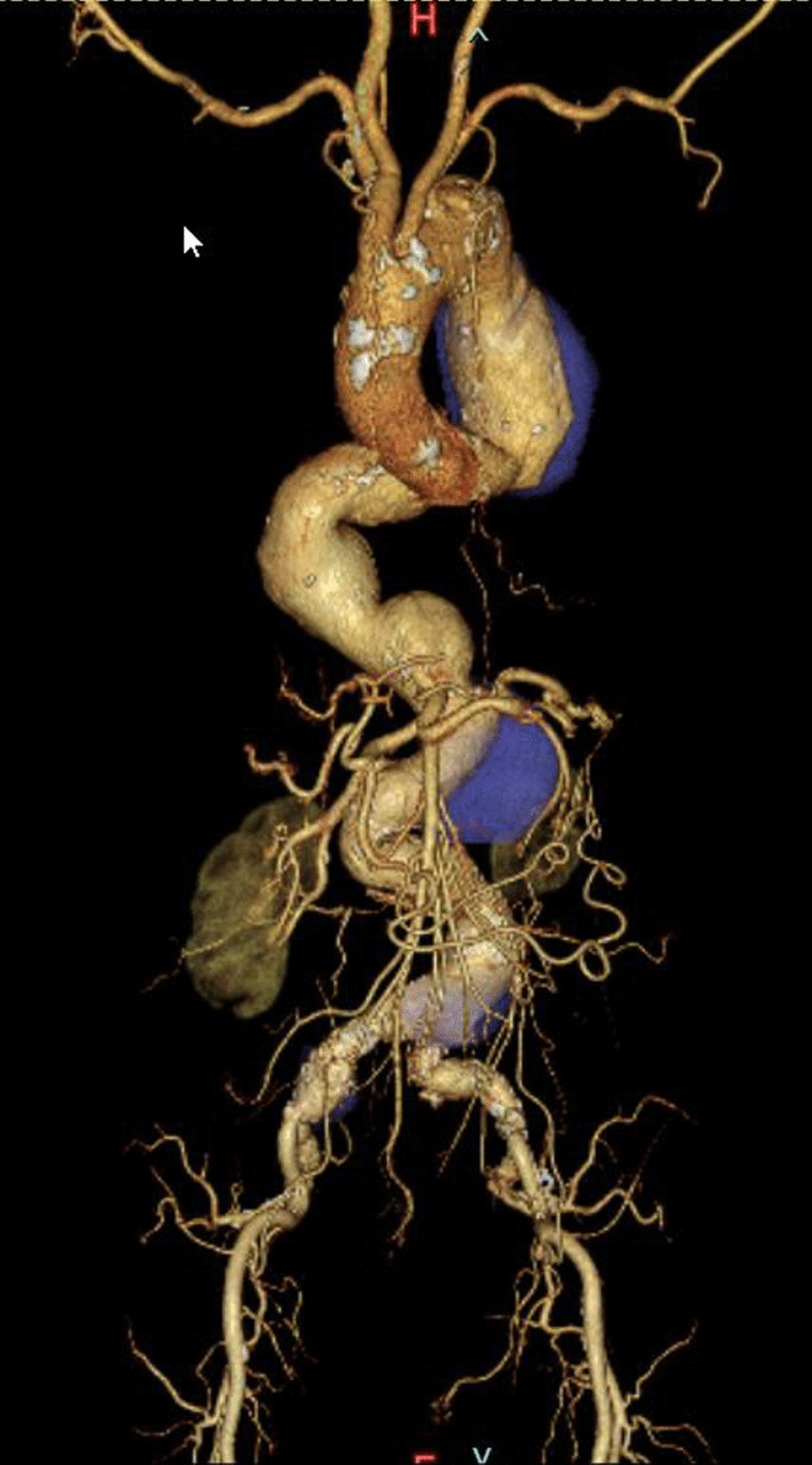
Preoperative computed tomography image. Computed tomography revealed a descending thoracic aortic aneurysm (65 mm in diameter), a thoracoabdominal aneurysm (54 mm in diameter), and an abdominal aortic aneurysm (49 mm in diameter)

**Fig. 2:**
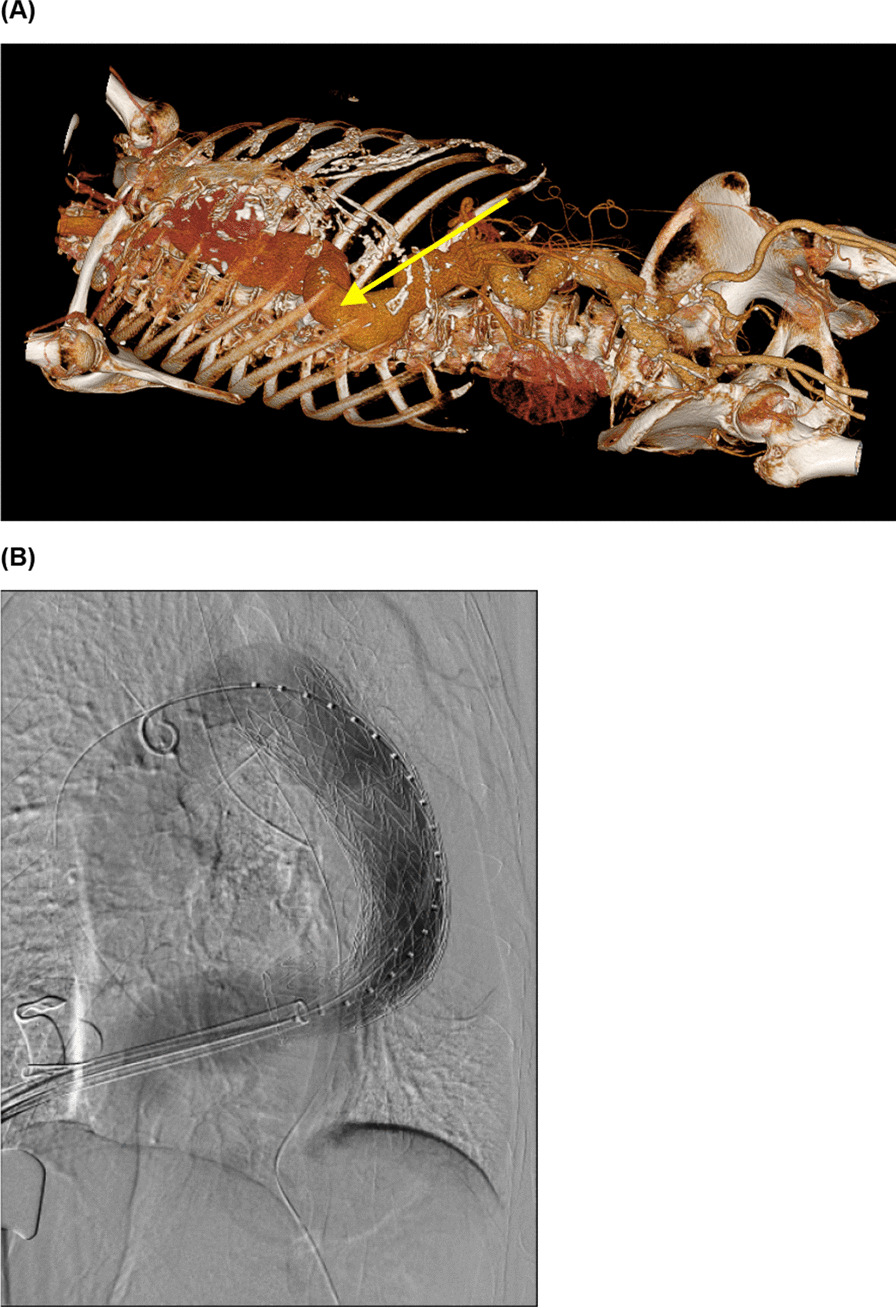
Approach to the descending aorta. **A** We approached the descending aorta with a right 6th intercostal thoracotomy and used it as an access route to perform TEVAR. **B** TEVAR was completed and no endoleak was found on confirmatory digital subtraction angiography

A 70-year-old woman with high blood pressure was referred to our hospital for cardiovascular surgery after a chest radiograph revealed an enlargement of the descending aorta. Computed tomography (CT) revealed the presence of multiple aneurysms with the following locations and diameters: (1) descending thoracic aorta [65 mm], (2) thoracoabdominal aorta [54 mm], and (3) abdominal aorta [49 mm] (Fig. 1). The patient was diagnosed with severe chronic obstructive pulmonary disease (COPD) preoperatively, based on a pulmonary function test that showed a forced expiratory volume of 47% per sec. Long-hour separate lung ventilation is required to perform one-stage thoracoabdominal replacement from descending aorta to thoracoabdominal aorta. But, our hospital anesthesiologist determined that long-term separate lung ventilation was not possible for this patient due to severe COPD. Therefore, we decided to perform TEVAR for the descending thoracic aortic aneurysm with the largest aneurysm diameter. TEVAR was scheduled for March 2021. The aorta (from the descending thoracic aorta to the abdominal) was very tortuous, hampering access to the surgical site from the common femoral artery. Furthermore, the diameter of the proximal landing zone was 40 mm, and the diameter of the distal landing zone diameter was 33 mm. It was necessary to use a 24Fr sheath, but the diameter of the left and right external iliac arteries was only 7 mm, and it was expected that it would be difficult to insert the sheath from the common femoral artery. The diameters of the bilateral subclavian artery and common carotid artery were both 7.5 mm or less, and it was expected that insertion of the 24Fr sheath would be difficult. Although we considered approaching from the abdominal aorta, this access route was difficult because the abdominal aorta and the both common iliac arteries also had an aneurysm. It was expected that the ascending aorta was highly calcified and it was difficult to insert the sheath. The access route that was not affected by the meandering of the aorta was considered to be the descending aorta with a right thoracotomy. Therefore, we decided to approach the descending aorta with a right 6th intercostal thoracotomy as an access route and perform TEVAR (Fig. 2A).

**Fig. 3:**
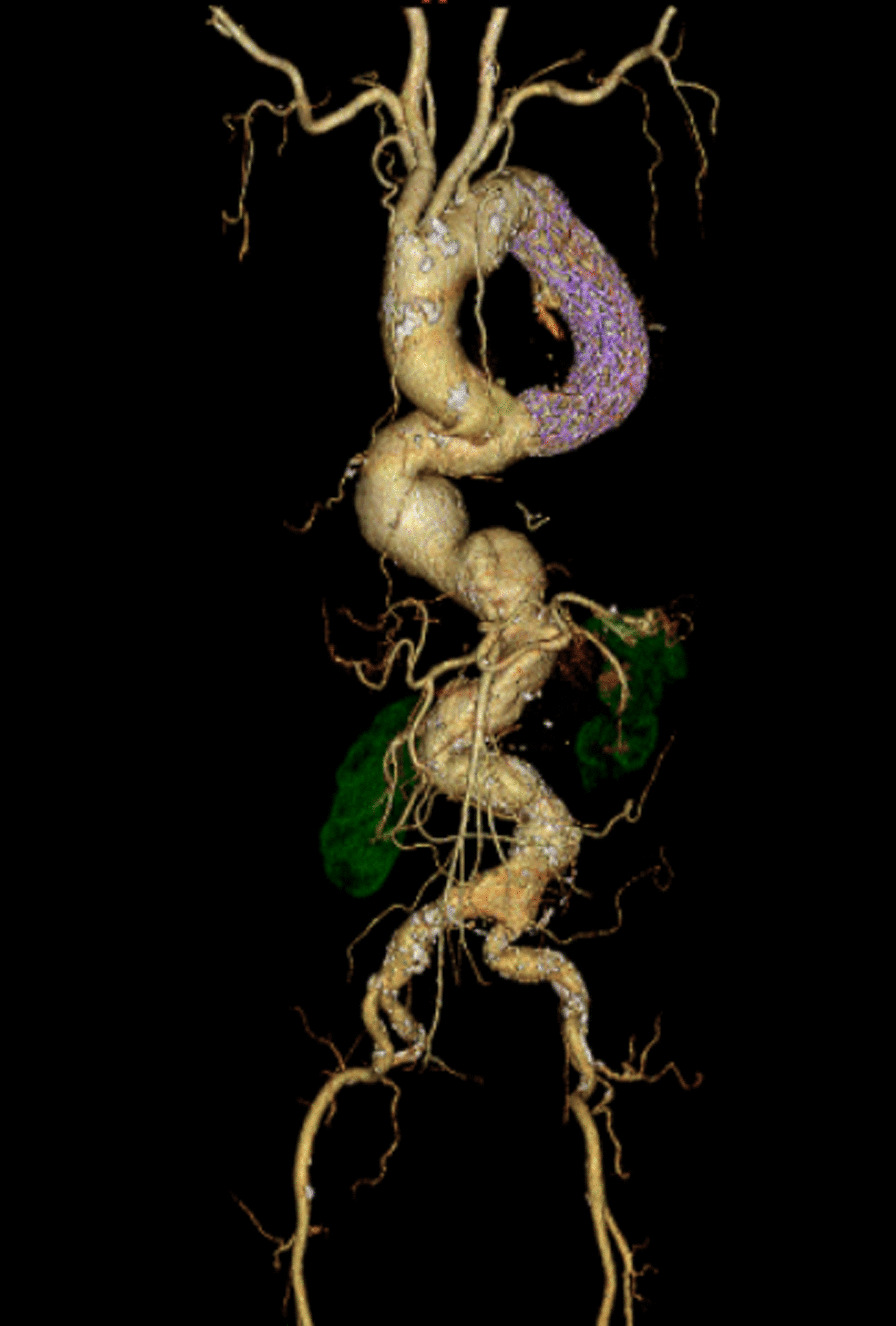
Postoperative computed tomography image. Postoperative contrast-enhanced CT images showed no endoleak

The patient was placed in the supine position with a 10° elevation of the superior right thorax for surgery under general anesthesia. We performed a thoracotomy about 7 cm in the right 6th intercostal space and exposed the descending aorta. Double purse-string sutures using felted 3–0 Ethibond were placed in the section of descending aorta to be punctured. A 6 French (Fr) sheath was inserted into the descending aorta. A marker pigtail catheter was inserted into the ascending aorta; the existing wire was replaced with a stiff wire and a 24 Fr delivery sheath (DrySeal, WL Gore, Flagstaff, AZ) was inserted into the distal aortic arch. At this time, separate lung ventilation was performed for a short period of time, but the oxygen saturation decreased significantly. The marker pigtail catheter was then advanced into the distal aortic arch and digital subtraction angiography (DSA) was performed to determine the necessary stent-graft size and placement location. The stent-graft (Gore cTAG 37–37-15, WL Gore, Flagstaff, AZ) was deployed at the periphery of the proposed indwelling stent site. After raising the sheath again, another stent-graft (Gore cTAG 45–45-15, WL Gore, Flagstaff, AZ) was inserted and deployed at the central region of the site. The stents overlapped by about 10 cm.The proximal landing zone and the stent-graft connection part were attached using a trilobe balloon catheter. We confirmed that there was no endoleak with DSA (Fig. 2B). The sheath was removed, and hemostasis was confirmed. A drain was placed in the right thoracic cavity. The operative time was 2.5 h. Intubation was maintained during a 2-h postoperative recovery period. The patient was extubated immediately upon admission to the intensive care unit. The patient was discharged from the intensive care unit 1 day after the operation; further postoperative recovery was uneventful. Postoperative contrast-enhanced CT images showed no complications (Fig. 3). The patient was discharged from the hospital on postoperative day 10.

## Discussion and conclusions

TEVAR has seen increased use as a procedure for treating descending thoracic aortic aneurysms and is the preferred procedure when anatomic requirements for its use are met [2, 3]. The common femoral artery is often selected as the access route for TEVAR. However, other access routes are required when: (1) the vascular diameter from the bifurcation of the common iliac artery to the common femoral artery is inadequate, (2) the arterial walls are poorly characterized, (3) arteries are tortuously shaped, or (4) access to the aorta is difficult. The external iliac artery and common iliac artery are also often used as access routes [1]. Additionally, there are reports of TEVAR via the abdominal aortic approach [4], ascending aortic approach [5, 6], apex approach [7], and carotid artery approach [8]. In our patient, the marked meandering from the descending thoracic aorta to the abdominal aorta made TEVAR with the normal approach problematic. In addition, none of the above alternative access routes could be selected as access routes due to calcification of blood vessels and small diameter of blood vessels. So, we decided to approach the descending aorta as an access route with a right thoracotomy Marked distortion of the path of the descending thoracic aorta allowed access to it with a right thoracotomy approach; therefore, that site was used for endovascular surgical access.

To the best of our knowledge, there are no reported cases of TEVAR performed by approaching the tortuous descending thoracic aorta through a right thoracotomy. I think that this treatment is less invasive than a descending thoracic aortic replacement or thoracoabdominal aortic replacement with long-hour separate lung ventilation. In this case, the patient had severe COPD, and only short-hour separate lung ventilation reduced blood oxygen saturation. It is speculated that long-hour separate lung ventilation would not have been possible. In addition, compared to the abdominal aortic approach, manipulation of the catheter was easier and operative time was shorter with this approach we used, since this latter route was not affected by twisting of the aorta.

In summary, our findings suggest that depending on the anatomical conditions, TEVAR can be performed by approaching the descending aorta with a right thoracotomy, if the common femoral artery approach is challenging.

## Data Availability

All data related to this study are stored in the electronic medical record of Sakai City Medical Center.

## Abbreviations

CT: Computed tomography
COPD: Chronic obstructive pulmonary disease
DSA: Digital subtraction angiography
TEVAR: Thoracic endovascular aortic repair
